# Localization, Concentration, and Transmission Efficiency of *Banana bunchy top virus* in Four Asexual Lineages of *Pentalonia* aphids

**DOI:** 10.3390/v5020758

**Published:** 2013-02-22

**Authors:** Shizu Watanabe, April M. Greenwell, Alberto Bressan

**Affiliations:** 1 Department of Plant and Environmental Protection Sciences, University of Hawaii at Manoa, Honolulu, HI 96822, USA; 2 Department of Molecular Bioscience and Bioengineering, University of Hawaii, Honolulu, HI 96822; USA; E-Mail: shizuw@hawaii.edu (S.W.); 3 NSF-Center for Integrated Pest Management, North Carolina State University, USDA APHIS PPQ office, Honolulu, HI 96850, USA; E-Mail: April.M.Greenwell@aphis.usda.gov (A.M.G.)

**Keywords:** *Pentalonia nigronervosa*, *Pentalonia caladii*, immunofluorescence localization, real time PCR

## Abstract

*Banana bunchy top virus* (BBTV) is the most destructive pathogenic virus of banana plants worldwide. The virus is transmitted in a circulative non-propagative manner by the banana aphid, *Pentalonia nigronervosa* Coquerel. In this work, we examined the localization, accumulation, and transmission efficiency of BBTV in four laboratory-established lineages of *Pentalonia* aphids derived from four different host plants: taro (*Colocasia esculenta*), heliconia (*Heliconia* spp.), red ginger (*Alpinia purpurata)*, and banana (*Musa* sp.). Mitochondrial sequencing identified three and one lineages as *Pentalonia caladii* van der Goot, a recently proposed species, and *P. nigronervosa*, respectively. Microsatellite analysis separated the aphid lineages into four distinct genotypes. The transmission of BBTV was tested using leaf disk and whole-plant assays, both of which showed that all four lineages are competent vectors of BBTV, although the *P. caladii* from heliconia transmitted BBTV to the leaf disks at a significantly lower rate than did *P. nigronervosa*. The concentration of BBTV in dissected guts, haemolymph, and salivary glands was quantified by real-time PCR. The BBTV titer reached similar concentrations in the guts, haemolymph, and salivary glands of aphids from all four lineages tested. Furthermore, immunofluorescence assays showed that BBTV antigens localized to the anterior midguts and the principal salivary glands, demonstrating a similar pattern of translocations across the four lineages. The results reported in this study showed for the first time that *P. caladii* is a competent vector of BBTV.

## 1. Introduction

Banana crop (*Musa* sp.) is cultivated in more than 120 countries and is an important staple crop for millions of people in several developing regions of the world [1]. Bunchy top disease, caused by *Banana Bunchy top virus* (BBTV) is one of the most important constraints on banana production in several countries of Asia, Africa, and the Pacific, including Hawaii [2,3,4]. Typical bunchy top symptoms include chlorosis of the leaf margins, narrowing and bunching of successive leaves, Morse code dashes, hooking along the midrib of the leaves, and dark green streaking of petioles [2]. The virus can move systemically to infect the entire banana corm and any new shoots derived from the corm [5]. In the field, plant symptoms are expressed 25-85 days after the initial infection with BBTV [6].

BBTV belongs to the family *Nanoviridae*, which contains at least eight formally recognized viral species within two genera, the *Nanovirus* and the *Babuvirus* [7]. The babuviruses include BBTV, *Abaca bunchy top virus* (ABTV), and *Cardamom bushy dwarf virus* (CBDV), whereas the genus *Nanovirus* includes viral species that infect legume crops such as *Faba bean necrotic yellows virus* (FBNYV) and *Faba bean necrotic stunt virus* (FBNSV). The BBTV genome is composed of up to 12 single-stranded circular DNA components of approximately 1 kb of nucleotides each. Each genomic component is separately encapsidated, within non-enveloped icosahedral particles of 18-20 nm in diameter [7]. All known nanoviridae are aphid-transmitted, and BBTV is specifically transmitted by *Pentalonia nigronervosa* Coquerel (Hemiptera, Aphididae), the banana aphid.

BBTV is transmitted through the aphid vector in a persistent circulative path [2,8]. The virus may be acquired and transmitted within a minimum acquisition access period (AAP) and inoculation access period (IAP) of 4 h and 15 min, respectively [9]. There is a detectable latent period for transmission in the aphid, estimated at 20-28 h [9,10]. *Banana Bunchy top virus* is retained in the vector after molting, and no transovarial transmission has been reported [8]. Previously, we have obtained direct evidence of the translocation path of BBTV through the aphid vector. Using immunofluorescence and immunocapture PCR procedures, we showed that BBTV antigens localize to the anterior midgut (AMG) and specific cells of the principal salivary glands (PSGs) [11]. In addition, we reported that BBTV rapidly translocates within the aphid vector; the viral particles first localize to the AMG, where they accumulate and are retained at higher concentrations than in either the haemolymph or the PSGs [12]. We have suggested two possible translocation paths; the first considers the movement of virions from the AMG to the PSGs through the haemolymph, while the second considers the direct translocation from the AMG to the PSGs because the tissues that form these organs can come into direct contact within the haemocoel [12].

These pieces of evidence show that BBTV, and potentially other members of the family *Nanoviridae*, uses a very different translocation path than the aphid-transmitted luteovirids [13,14]. Indeed, the hindguts rather than the AMG have been shown to be the first site of internalisation of the luteovirids, including *Barley yellow dwarf virus* (*Luteoviridae, Luteovirus*; BYDV), *Cereal yellow dwarf virus* (*Luteoviridae, Polerovirus*; CYDV), *Beet western yellow virus* (*Luteoviridae,* *Polerovirus*; BWYV) and *Potato leafroll virus* (*Luteoviridae, Polerovirus*; PLRV); the posterior midgut has been shown to be the site of internalisation, in addition to the hindgut, for the latter two poleroviruses [15,16,17]. Unlike BBTV, the luteovirids cross the accessory salivary glands tissues of competent aphid vectors.

*Pentalonia nigronervosa* was previously reported to contain two forms: “typica” and “caladii” [18]. Based on morphology and molecular data, Foottit and colleagues (2010) have recently re-classified *P. nigronervosa* f. *caladii* as a new species, *Pentalonia caladii* (Hemiptera, Aphididae), as originally proposed by van der Goot (1917). Although some sexual forms of *Pentalonia* aphids have been reported in northeast India and Nepal [19], both *P. nigronervosa* and *P. caladii* are believed to reproduce exclusively asexually in most subtropical and tropical regions, including the Pacific. Both species have the potential to exploit common hosts [4,20]; however, in nature, they differ in their ranges. *Pentalonia nigronervosa* primarily colonizes banana (*Musa* spp.) and taro (*Colocasia esculenta*) plants, whereas *P. caladii* chiefly colonizes ginger (*Zingiber officinale*, *Alpinia purpurata*, *Hedychium coronarium*), heliconia (*Heliconia* spp.) and taro plants [21]. Recently, a population genetics study of *Pentalonia* aphids using microsatellite markers found that, in Hawaii, *P. nigronervosa* and *P. caladii* include distinct genotypes, demonstrating the occurrence of intraspecific genetic variation [22].

While aphids display intra- and interspecific variation in luteovirid vector competence [23,24], it is currently unknown whether distinct genotypes of *Pentalonia* spp. exhibit variations in the transmission competency of BBTV. In addition, although transmission experiments have failed to demonstrate that taro and ginger plants (*A. purpurata*), both of which are among the preferred hosts of *P. caladii*, can be infected by BBTV [25], the extent to which *P. caladii* is a competent vector of BBTV and may play an active role in the epidemiology of banana bunchy top disease is still unclear.

In this study, we have tested the competency of 4 asexual lineages of *Pentalonia* aphids collected from taro, red ginger, heliconia, and banana plants for the transmission of BBTV. In addition, we examined the concentration and localization of BBTV in these lineages using real time PCR and immunofluorescence assays. Our work has two goals: first, to determine the competence of *P. caladii* in the transmission of BBTV, and second, to identify potential cellular barriers of BBTV transmission.

## 2. Results and Discussion

### 2.1. Aphid identification

In the spring of 2010, we collected *Pentalonia* aphids from four different plants, banana, red ginger (*A. purpurata*), heliconia and taro, on the island of Oahu, Hawaii, and we established four greenhouse-reared lineages (Table 1). Based on a BLAST search of GenBank using the Cytochrome Oxidase I gene sequence fragments obtained, the aphid lineage isolated from the banana was identified as *P. nigronervosa*, whereas the other lineages, originally collected from ginger, heliconia, and taro, were identified as *P. caladii* (Table 1). Based on 9 microsatellite markers, each lineage was assigned to a distinct multilocus genotype (MLG) (Table 1).

**Table 1:** *Pentalonia* aphids collection and identification as species and as Multi Locus Genotype (MLG).

Host Plant	Collection Date	Coordinates	Species	MLG
Banana	7 April 2010	N 21 16.995 W 157 42.025	*P. nigronervosa*	P_1_
Ginger	7 May 2010	N 21 18.883 W 157 48.422	*P. caladii*	P_2_
Heliconia	7 May 2010	N 21 19.033 W 157 49.919	*P. caladii*	P_3_
Taro	7 May 2010	N 21 20.235 W 157 43.317	*P. caladii*	P_4_

### 2.2. BBTV quantification

**Figure 1:**
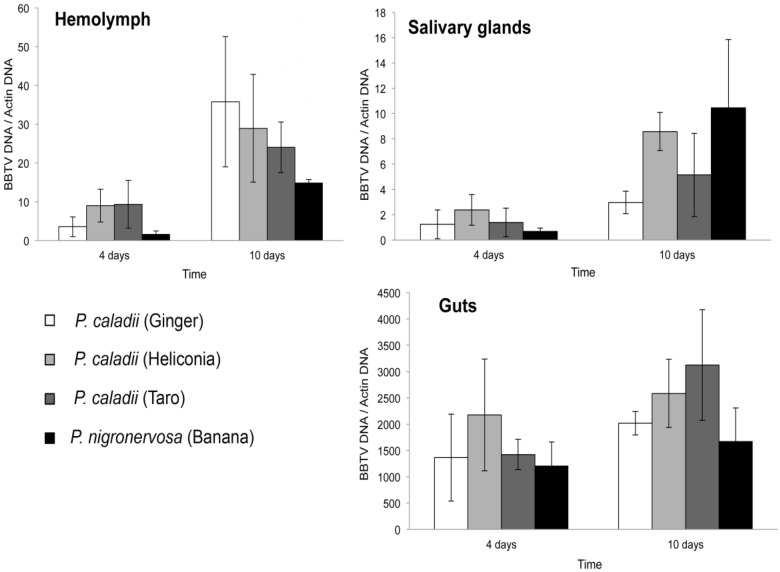
Average concentration and standard error of *Banana bunchy top virus* (BBTV) in the gut (a), salivary glands (b), and haemolymph (c) of *Pentalonia nigronervosa* and *P. caladii* derived from three different host plants: taro, heliconia, and red ginger.

The titer of BBTV-S, which contains an ORF encoding the coat protein [26], was quantified in aphid tissues using real-time PCR assays. Figure 1 shows the average concentration of BBTV relative to the aphid actin gene in the gut, salivary glands, and haemolymph for the four aphid lineages fed from BBTV-infected banana plants for 4 or 10 days. Consistent with previous results [12], the relative amounts of BBTV seemed to be much greater in the gut tissues than in the salivary glands and haemolymph in all four lineages (Fig. 1).

Irrespective of the time of aphid feeding on the infected plants, there were no significant differences in the concentration of BBTV in the gut, salivary glands, or haemolymph across the four lineages (Kruskal-Wallis, *Ps* > 0.05). The concentration of BBTV increased significantly in the haemolymph (Mann-Whitney U = 15, *P* = 0.001) and salivary glands (Mann-Whitney U = 17, *P* = 0.002) of aphids that fed for 4 or 10 days on virus-infected plants. However, we did not observe an increase of BBTV in the gut (Mann-Whitney U = 44, *P* = 0.112), suggesting that most of the retention sites were saturated after the first 4 days of feeding.

The average titer of BBTV in the salivary glands increased by 15.5-, 3.68-, 3.61-, 2.39-fold in *P. nigronervosa* and *P. caladii* from taro, heliconia, and ginger, respectively (Fig. 1). The average titer of BBTV in the haemolymph was also increased by 8.9-, 2.6-, 3.2-, 10.1-fold in *P. nigronervosa* and *P. caladii* from taro, heliconia, and ginger, respectively (Fig. 1). The relative concentration of BBTV in the guts increased by 1.4-, 2.2-, 1.2-, and 1.5-fold in *P. nigronervosa* and *P. caladii* from taro, heliconia, and ginger, respectively (Fig. 1). Those averages were not significantly different (Kruskal-Wallis, *Ps* > 0.05).

### 2.3 BBTV localization

**Figure 2:**
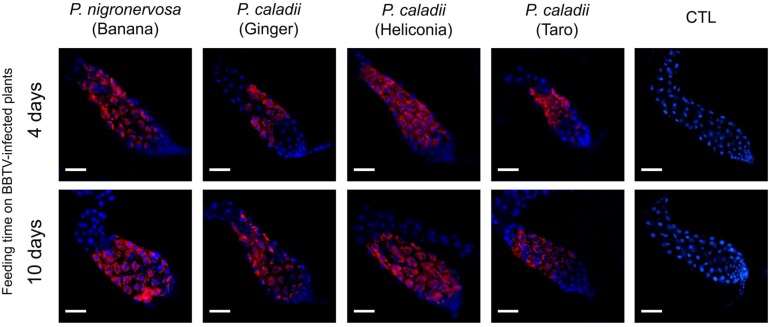
Immunofluorescent localization of *Banana bunchy top virus* (BBTV) in paraformaldehyde-fixed anterior midguts (AMGs) of *Pentalonia nigronervosa* and *P. caladii* aphids derived from three different host plants: taro, heliconia, and red ginger. BBTV antigens were labeled with mouse monoclonal antibodies and detected with Alexa Fluor 555 conjugated secondary antibodies (red). Cell nuclei were stained with DAPI (blue). CTL, control. Scale bar = 100 µm.

**Figure 3:**
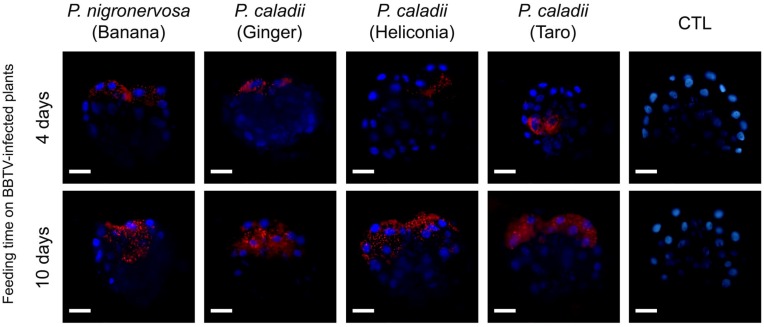
Immunofluorescent localization of *Banana bunchy top virus* (BBTV) in paraformaldehyde-fixed principal salivary glands (PSGs) from *Pentalonia nigronervosa* and *P. caladii* derived from three different hosts: taro, heliconia, and red ginger. BBTV antigens were labeled with mouse monoclonal antibodies and detected with Alexa Fluor 555 conjugated secondary antibodies (red). Cell nuclei were stained with DAPI (blue). CTL, control. Scale bar = 50 µm.

In parallel with real-time PCR, we performed immunofluorescence assays to determine the localization of BBTV antigens. The localization of BBTV in the gut tissues appeared to be similar across the different aphid lineages tested over time (Fig. 2). After 4 or 10 days, labeling was observed in the cytoplasm surrounding the cell nuclei of the AMGs (Fig. 2). Confirming previous research [12], we did not observe BBTV labeling in the accessory salivary glands of any aphid lineage (data not shown). Detectable levels of labeling were observed in the PSGs from all aphid lineages, in which distinct puncta were observed after 4 days of aphid feeding on BBTV-infected plants (Fig. 3), and the labeling became more prominent after 10 days (Fig. 3). However, the degree of labeling varied between individual aphids, and a large proportion of salivary glands did not show obvious labeling patterns (Table 2). Immunofluorescence assays corroborate the results obtained by using real time PCR, which suggest the concentration of BBTV is much greater in the guts than in the salivary glands (Fig. 2, 3).

**Table 2:** Detection of *Banana bunchy top virus* within four different aphid lineages fed on BBTV-infected plants for 4 or 10 days using immunofluorescence assays. Images of the labeling of the anterior midguts and principal salivary glands are shown in Figure 2, Figure 3, respectively.

Host plant	Species	MLG	4 days of feeding on BBTV-infected plants^1^		10 days of feeding on BBTV-infected plants^1^	
			Anterior midgut	Principal salivary glands	Anterior midgut	Principal salivary glands
Banana	*P. nigronervosa*	P_3_	15/27 (0.56)	3/17 (0.18)	21/25 (0.84)	12/24 (0.5)
Heliconia	*P. caladii*	P_2_	8/21 (0.38)	2/17 (0.12)	34/46 (0.74)	19/33 (0.58)
Ginger	*P. caladii*	P_4_	8/27 (0.30)	3/17 (0.29)	8/19 (0.42)	1/11 (0.09)
Taro	*P. caladii*	P_7_	10/19 (0.53)	1/12 (0.08)	5/17 (0.29)	5/18 (0.28)

^1^ Number of dissected organs labeled with the virus / total number of organs tested. Proportions are reported in parentheses.

### 2.4 Transmission assays

**Figure 4:**
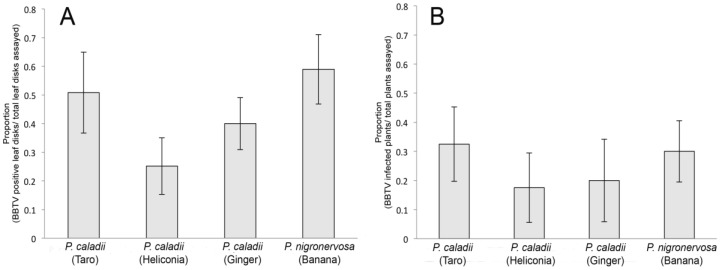
Transmission of *Banana bunchy top virus* (BBTV) by *Pentalonia nigronervosa* and *P. caladii* derived from taro, heliconia, and red ginger, as measured by (A) leaf disk assays and (B) whole plant assays.

We examined the transmission of BBTV across the four aphid lineages using whole plant assays. Using groups of 3 aphids per plant, we demonstrated that the four aphid lineages were all competent vectors (Fig. 4) and transmitted BBTV with similar efficiencies (F_3, 12 _= 0.35, *P* = 0.78) (Fig. 4B). To obtain a more precise assessment of transmission competency, the transmission of BBTV was additionally analyzed using leaf disk assays and individual aphids. In the leaf disk assays, the transmission efficiency varied significantly among lineages (F_3, 24 _= 3, *P* = 0.049). In fact, the Tukey tests revealed that transmission rate of *P. caladii* derived from heliconia was significantly lower than that of *P. nigronervosa* (Fig. 4A) but not significantly different from *P. caladii* derived from taro and ginger.

Different biotypes of hemipteran insects have shown differences in the transmission efficiency of circulatively transmitted plant viruses. For instance, experimental evidence has shown that the greenbug *Schizaphis graminum* (Hemiptera, Aphididae), the bird cherry-oat aphid *Rhopalosiphum padi* (Hemiptera, Aphididae) and the English grain aphid *Sitobion avenae* (Hemiptera, Aphididae) display intra-specific variation in the transmission efficiency of BYDV-MAV or -PAV isolates [27,28]. The whitefly *Bemisia tabaci* (Hemiptera, Aleyrodidae) is the exclusive vector of the *Begomovirus* genus, which lists several viral species and strains [29]. *Bemisia tabaci* belongs to a complex of genetic species, referred as biotypes [30], which seem to differ in the competences of transmitting the Begomoviruses. For instance, Gottlieb et al. (2010) showed that the B biotype of *Bemisia tabaci* transmits *Tomato yellow leaf curl virus* (*Geminiviridae,* *Begomovirus*; TYLCV) more efficiently than the Q biotype. Interestingly, differences in TYLCV transmission seem to be related to the presence of specific secondary bacteria endosymbionts that facilitate TYLCV transmission by producing GroEL proteins that specifically bind to the viral particles and inhibit their proteolysis in the vector haemolymph. 

Several aphid-transmitted luteovirids are vectored with a high degree of specificity [14,24]. During the process of transmission, luteovirids cross two epithelial cell layers in the aphid vector: the gut, at the level of the hindgut and posterior midgut, and the accessory salivary glands [15,31]. Because non-transmissible luteovirids may fail to cross either of these cellular epithelia in non-aphid vectors, these tissues have been referred to as ‘transmission barriers’ [31]. For example, transmission electron microscopy has shown that, in non-vector aphids, BYDV-MAV can be detected at the basal lamina of the hindgut, but no viral particles are detected in the cytoplasm of the cells that form the accessory salivary glands, suggesting that the virus is not transmissible because it cannot cross the basal lamina and/or basal plasmalemma of the salivary glands [16,32]. This study suggests that there are no major cellular barriers for BBTV transmission in the 4 aphid lineages as the concentration and the pattern of viral particles localization in the AMGs and in the PSGs were similar. Therefore, we suggest the low transmission rates of BBTV by *P. caladii* from heliconia may result from differences in the feeding behaviour of that aphid lineage on banana tissues.

As reported in this and previous studies [11,12], the process of BBTV translocation has more similarities with the begomoviruses rather than the luotevirids. In fact, the internalisation of the begomoviruses occurs through the anterior midgut and filter chamber [33,34,35] of *B. tabaci*. Viral particles are then released into the hemolymph and are finally transmitted to plants following penetration of the PSGs [33,34]. A similar pattern of translocation has been described for another circulatively transmitted geminivirus, *Maize streak virus* (*Geminiviridae*, *Mastrevirus;* MSV), through the leafhopper vector *Cicadulina mbila* (Hemiptera, Cicadellidae) [36]. 

While there is little information available on the cellular process of internalization of begomoviruses in *B. tabaci*, it has been proposed that luteovirid uptake occurs via clathrin-mediated endocytosis, which involves the formation of clathrin-coated invaginations on the plasma membrane that recruit cell-surface receptor(s) [13]. Several aphid proteins potentially involved in virus internalisation have been identified in the green peach aphid *Myzus persicae* (Hemiptera, Aphididae) and *S. graminum*, namely actin, GAPDH, Rack-1, cyclophilin, and luciferase [37,38]. In addition, a transcriptomic analysis of aphid intestinal genes carrying the *Pea enation mosaic virus* (*Luteoviridae*, *Enamovirus*; PEMV) showed limited levels of down- and up-regulation, suggesting that the virus hijacks a constitutive endo-exocytosis mechanism without significantly perturbing aphid cell metabolism [31,39]. Recently, several additional marker proteins associated with luteovirid transmission in the aphid vector and its endosymbiont *Buchnera aphidicola* have been identified through proteomic analysis coupled with differential gene electrophoresis (DIGE) assays [40,41]. 

To date, the cellular mechanisms of nanovirid internalization in aphid cells have not been characterized. However, the involvement of a virus-encoded helper factor has been suggested based on evidence that purified *Nanovirus* particles are not aphid-transmissible and the successful completion of the transmission by aphids through transmission complementation assays [42]. 

## 3. Experimental Section

### 3.1 Aphid collection, rearing and identification

To establish the four greenhouse-reared linages, one apterous female from each colony was placed on a detached host plant leaf within a self-watering acrylic cage [43]. The first instar nymphs produced by each aphid were transferred to potted individual banana plants, with the exception of the lineage from the taro plant, which reproduced very slowly on banana plants and therefore was reared on taro, its original host. All plants were contained within poly-organza mesh fabric cages (Super Poly Organza, Hyman Hendler and Sons, Los Angeles, CA) and maintained in a greenhouse (26 ± 5 °C). The colonies were maintained by periodically transferring aphid-infested leaves to new un-infested plants.

The four aphid lineages were identified at the species level by sequencing of a portion of the Cytochrome Oxidase subunit I (COI) gene as described by Foottit *et al.* (2010) (Table 1). Asexual lineages were further characterized at the intraspecific level using nine microsatellite markers (M62, S24, Ago66, S17b, S16b, S23, AF169, AF-4, and AF-1) and assigned to multilocus genotypes (MLGs) using GenoType and GenoDive software, as previously described [22,44]. Aphids were used in experiments after a rearing period of approximately 18 months.

### 3.2 Plant and virus maintenance

Banana plants (cv Williams) were generated from tissue culture by the Seed Lab of the University of Hawai’i at Manoa, College of Tropical Agriculture and Human Resources (CTAHR). Taro plants were obtained from a local nursery at Waimanalo on the island of Oahu (Hawaii; USA). All plants were grown in pots containing Sunshine Mix 4 (Sun Gro Horticulture Distribution, Vancouver, Canada), vermiculite and perlite at ratio of 3:2:1 and were kept in a greenhouse with natural light and a temperature range of 26 ± 5 °C.

A strain of BBTV collected in August 2007 from a field-infected banana plant on the Island of Oahu (Hawaii; USA) was transmitted to potted micropropagated banana plants using an aphid vector. The virus was characterized with TAS ELISA (Agdia Inc., Elkhart, IN, USA) and PCR assays using primer pairs for the detection of 6 BBTV DNA components [45] according to previously described methods [46,47]. Sequencing of the partially amplified genomic components showed that the sequences of this strain were nearly identical to those in previous works [46,47] and confirmed that BBTV from Hawaii clusters with the Middle Eastern-South Pacific clade of BBTV [48].

### 3.3 Transmission assays

Transmission assays were conducted to examine whether all four *Pentalonia* lineages were capable of transmitting BBTV to potted banana plants. Fourth instar aphids of each lineage were sampled from the rearing colonies and allowed to feed on BBTV-infected plants for a 4-day acquisition access period (AAP). At the end of the AAP, groups of three aphids of each lineage were transferred to healthy banana plants at the 3-5 leaf stage for an inoculation access period (IAP) of 7 days. Ten plants were assayed per treatment, and the experiment was replicated four times. At the end of the IAP, aphids were removed from the plants, which were then sprayed with an aqueous solution containing 0.0120% Imidacloprid (Bayer Rose and Flower Insect Killer, Research Triangle Park, NC) and transferred to insect-proof cages (BioQuip; 35.5 × 35.5 × 70 cm) in the greenhouse. Plants were visually inspected on a weekly basis for symptom development for a period of 85 days; those plants not displaying typical bunchy top symptoms were further analyzed for the presence of BBTV by diagnostic PCR as described in Watanabe and Bressan (2012).

To obtain a more precise assessment of transmission competency, the transmission of BBTV was additionally analyzed using leaf disk assays and individual aphids as previously described [11]. Leaf disk chambers were generated by transversely cutting 15-ml Falcon conical tubes 15 mm below the lid. Leaf disks of 13 mm in diameter were excised from healthy banana plant leaf midribs. A 1 ml aliquot of 1% melted agar solution was poured into the cap, and a leaf disk was placed on top of the agar before it solidified. The aphids were allowed to feed on BBTV-infected plants for 4-day AAP and then individually transferred to healthy banana leaf disks. The tops of the chambers were sealed with stretched Parafilm^®^. At the end of the 2-day IAP, aphids were removed from the chambers, and the leaf disks were kept in the agar, inside a sealed humid chamber to prevent dehydration, for another four days to allow the virus to replicate locally [11]. Transmission assays were carried out in a growth chamber at 25 °C with a photoperiod of 12: 12 (light: dark). Leaf disks were then removed from the chamber, thoroughly washed with dish soap on both sides of each leaf, and rinsed in water. The DNA was extracted from individual leaf disks using the cetyltrimethylammonium bromide (CTAB) method [41]. The presence of BBTV was analyzed by nested PCR as described in Watanabe and Bressan (2012). The first round of amplification was performed using 1 μl of DNA in 25 μl of master mix containing 5 pmol of primers 73F (5′-GGCTTTATCCAGAAGACCAA-3′) and 73R (5′-CCGTATCATGTATATTTGTTT-3′) to specifically detect BBTV-S. The PCR products obtained from the first round of amplification were diluted 30-fold, and 1 μl of the diluted product was used as the template for a second round of amplification, with a master mix containing 5 pmol of each primer CPXI.PRI and BBTV3C.EXP [49] to amplify an internal portion of the BBTV-S produced by the direct PCR. Bands were visualized in a 1.2% agarose gel. Leaf disk transmission experiments were replicated 3 times. 

For both plant and leaf disk assays, BBTV transmission was expressed as the proportion of BBTV-infected leaf disks or plants divided by the total number of aphid-inoculated leaf disks or plants across the four lineages. Proportions were arcsine transformed and compared using one-way analysis of variance (ANOVA) followed by Tukey’s test for post-hoc comparisons. Statistical analyses were performed in SigmaStat Version 12.0 (SPSS, Inc., Chicago, IL).

### 3.4 Aphid dissection

Aphids were dissected under a stereomicroscope to examine the localization and titre of BBTV in the haemolymph, salivary glands, and gut tissues. Briefly, individual aphids were immobilized on the surface of a dissecting chamber (Electron Microscopy Sciences, Hatfield, PA, USA) and immersed in approximately 200 μl of phosphate-buffered saline (PBS; 137 mM NaCl, 2.7 mM KCl, 10 mM Na_2_HPO_4_, and 2 mM KH_2_PO_4_, pH 7.4). The PBS-immersed aphids were then dissected with a polytetrafluoroethylene (PTFE)-coated stainless steel blade (Ted Pella Inc., Redding, CA, USA) by cutting the aphid bodies behind the compound eyes and allowing both the salivary duct and the foregut to separate from the stylets. The haemolymph released after decapitation was sampled with a glass capillary tube. The haemolymph of the banana aphid is red, whereas the internal organs are white-translucent, thus allowing the selective sampling of the haemolymph. The haemolymph sampled was transferred into a 1.5 ml microcentrifuge tube. Using small pins, the entire digestive tract and salivary glands were then dissected and removed from the aphid body. The organs were washed multiple times in PBS and processed for immunofluorescence localization and real-time PCR assays. The haemolymph samples were evaluated with real-time PCR assays only.

### 3.5 BBTV quantification

To quantify the relative concentration of BBTV, we first isolated DNA from the aphid tissues using a QIAamp DNA mini kit (Qiagen Inc., 370 Valencia, CA, USA). Briefly, the dissected guts, salivary glands, and haemolymph from 3 aphids were pooled into individual 1.5 ml ultracentrifuge tubes containing 180 μl of lysis buffer and processed according to the manufacturer’s instructions. The DNA was eluted with 100 μl of ultrapure water into clean 1.5 ml ultracentrifuge tubes. We used a relative real-time PCR method to target the BBTV DNA genomic component S and, as an internal reference, the banana aphid actin gene [12,50]. Briefly, PCR was performed with SYBR^®^ Green master mix (Qiagen Inc., Valencia, CA, USA), 500 nM of each primer (Table 3), and DNA in a final volume of 20 μl. Each DNA sample was analyzed in duplicate with a Rotor-GeneTM 6000 Thermal Cycler. 

**Table 3:** Sequences of the primers used for real-time PCR [12]

Target gene	Primer name	Sequence (5’ à3’)	Length (nt)	Product size (bp)
Actin	ActPef	CGGTGATTTCCTTTTGCATT	20	104
	ActPer	GTGTGACGTTGACATCAGAAAAG	23	
BBTV	BBTVSf	TGGGCTAATGGATTGTGGAT	20	91
	BBTVSr	CGCCTGTTTTTGGTCTTCTG	20	

For each sample tested, the relative amount of BBTV DNA was normalized to the amount of actin DNA using the following equation: ECt (actin)/ECt (BBTV), where E = the PCR efficiency of a given primer pair, and Ct = threshold cycle, which was automatically calculated by the IQ software (Rotor-Gene 6000 Series Software Version 1.7, Corbett Research, San Francisco, CA). The amplification efficiency was determined via linear regression through the amplification of serially diluted DNA extracts. Efficiency values were calculated with the following formula: 10^(–1/slope)^. Because E = 2.00 was achieved for both the BBTV and actin primer pairs, we adopted a simplified equation, 2 ^Ct (actin – BBTV)^, to calculate the relative abundance of the BBTV DNA in each sample. For each time point, we tested the DNA extracted from the guts, salivary glands and haemolymph of 18 aphids from each clonal lineage. The differences in the BBTV DNA concentrations among the four lineages were evaluated with the Kruskal-Wallis test followed by the Mann-Whitney for pairwise comparisons.

### 3.6 BBTV localization

Dissected organs were first allowed to fully adhere to the slide surface and then fixed in PBS containing 4% paraformaldehyde for 1 h. After extensive washing with PBS to remove the fixative, the slides were incubated with PBS containing 1% Triton X-100. The samples were then incubated for an additional hour in a blocking solution of PBS supplemented with 10% normal goat serum. Anti-BBTV mouse monoclonal antibodies (Ig/A24876 and Ig/A24877) were diluted 1:500 in PBS containing 1% normal goat serum and added to the slides, which were then incubated overnight at 4 °C. The slides were subsequently washed three times in PBS and incubated for 45 min with Alexa Fluor 555-conjugated goat anti-mouse IgG (Invitrogen, Carlsbad, CA), diluted 1:500 in PBS. The slides were washed three times in PBS, rinsed in water, and mounted in ProLong^®^ gold anti-fade mounting medium containing 4′,6-diamidino-2-phenylindole (DAPI; Invitrogen, Carlsbad, CA, USA) to counterstain the cell nuclei. The slides were visualized under an Olympus BX-51 epifluorescence microscope with an Optronics MacroFire digital camera.

## 4. Conclusions

In this study, we performed transmission assays combined with real-time PCR and immunofluorescence to examine the extent to which the accumulation, tropism, and transmission competencies of BBTV varied across four asexual lineages of *Pentalonia* aphids. The aphid lineage established from the banana plant was identified as *P. nigronervosa*, whereas the lineages originally collected from ginger, heliconia, and taro plants were all identified as *P. caladii*. Each lineage was assigned to a distinct MLG based on nine microsatellite markers. The results obtained showed that the four lineages are competent vectors of BBTV, although the *P. caladii* derived from heliconia transmitted BBTV to the banana leaf disks at a very low rate. As revealed by real-time PCR and immunofluorescence assays, BBTV reached higher concentrations in the gut tissues than in either the haemolymph or the salivary glands. In addition, immunofluorescence assays demonstrated that the localization of the BBTV antigen was similar across the four aphid lineages, suggesting that the virus has a similar translocation path in all four lineages.

The results reported in this study showed for the first time that *P. caladii* is a competent vector of BBTV. *Pentalonia caladii* colonises tropical flowers and ornamental plants including ginger, heliconia and taro [19,51]. In addition, ginger, heliconia and taro plants often grow in close proximity to banana fields in Hawaii. Transmission experiments have failed to demonstrate that taro and ginger plants serve as hosts for BBTV [25], and the *P. caladii* aphids colonising those plants may not play an active role in the transmission of BBTV, even when the aphids disperse from their primary hosts to banana plants. However, as shown in this work, *P. caladii* is a competent vector of BBTV, and is capable of acquiring the virus from infected banana plants and transmitting it to other banana plants. This later possibility implies that *P. caladii* aphids may extensively colonise banana crops. The current information available from population genetic studies and life table analysis suggests that *P. caladii* displays a strong host plant preference [21,22,52]. Therefore, banana-banana transmission of BBTV by *P. caladii* may not be very common; however, more extensive field surveys and host preference studies are needed to test this hypothesis. 

In summary, the data presented here, along with previous research [23,24,52], suggest that *P. nigronervosa* is likely to have a greater epidemiological importance in the spreading of BBTV than *P. caladii*; although *P. caladii* is a competent vector, its ecology may limit its vector activity in the field.

In the perspective of basic research, additional study may focus on the cellular mechanisms that are involved in the internalization of BBTV viral particles through the epithelial tissues (AMG and PSGs) of the aphid vectors, and on the possible interaction of BBTV with GroELs proteins produced by endosymbiotic bacteria that are harboured in *Pentalonia* aphids haemocoel [53].
